# Response to Controlled Ovarian Stimulation Is Not Impaired in Young Patients with a Sarcoma: Results from a Monocentric Case–Control Study

**DOI:** 10.3390/cancers15123141

**Published:** 2023-06-11

**Authors:** Raffaella Cioffi, Luca Pagliardini, Antonio Quartucci, Enrico Papaleo, Valeria Stella Vanni, Salvatore Provenzano, Rossella Bertulli, Massimo Candiani, Giorgia Mangili

**Affiliations:** 1 Department of Obstetrics and Gynaecology, San Raffaele Scientific Institute, 20132 Milan, Italy; pagliardini.luca@hsr.it (L.P.); quartucci.antonio@hsr.it (A.Q.); papaleo.enrico@hsr.it (E.P.); vanni.valeria@hsr.it (V.S.V.); candiani.massimo@hsr.it (M.C.); mangili.giorgia@hsr.it (G.M.); 2 Department of Brain and Behavioral Sciences, University of Pavia, 27100 Pavia, Italy; 3 Medical Oncology Department, Fondazione IRCCS Istituto Nazionale dei Tumori, 20133 Milan, Italy; salvatore.provenzano@istitutotumori.mi.it (S.P.); rossella.bertulli@istitutotumori.mi.it (R.B.)

**Keywords:** fertility preservation, oocyte cryopreservation, ovarian stimulation, oncofertility, sarcoma

## Abstract

**Simple Summary:**

The impact of a cancer diagnosis on fertility is debated. Some authors suggested a detrimental effect on ovarian function and response to ovarian stimulation in patients suffering from breast cancer and hematological malignancies. Sarcomas are rare but relatively common in young women, and usually, their treatment requires the use of highly gonadotoxic chemotherapy regimens. The aim of this study was to evaluate the performance of patients with sarcoma during controlled ovarian stimulation for oocyte cryopreservation, compared to age-matched healthy controls. Our results indicate that patients with a sarcoma diagnosis can expect good oocyte retrieval outcomes after controlled ovarian stimulation. Therefore, oocyte cryopreservation should be recommended to these patients, whenever possible, before the beginning of gonadotoxic treatments.

**Abstract:**

Sarcomas are relatively common in the young and their treatment can impair fertility. Fertility preservation can be achieved via the cryopreservation of gametes after controlled ovarian stimulation before cancer treatment. A reduced response to hormonal stimulation in patients suffering from certain types of malignancy is reported. The purpose of this study was to assess the performance of oocyte cryopreservation in patients with sarcoma by comparing their outcomes with those of a population without cancer. Patients were matched by age with control women undergoing hormonal stimulation for isolated male factor infertility. The population included 84 women with a sarcoma and 355 controls. In the final analysis, 37 patients with sarcoma were matched in a 1:3 ratio with 109 healthy controls. Patients with sarcoma were generally younger and were stimulated with lower FSH doses. They did not perform worse than controls during stimulation, with an average retrieval of 10.6 oocytes vs. 8.1 in the controls. Linear regression on the number of retrieved mature oocytes confirmed that patients with sarcoma performed comparably to controls. In conclusion, patients with sarcoma can expect retrieval outcomes comparable to those of patients without cancer.

## 1. Introduction

Unlike most types of cancer, and although they represent only 1% of all adult solid cancers, sarcomas frequently occur in the young, accounting for 20% of childhood cancers and 10% of adolescent and young adult cancers [1]. The most frequent histologies in this age group include liposarcoma, Ewing sarcoma, epithelioid, clear cell, and synovial sarcoma [2]. Treatment normally requires a combination of different strategies including multi-agent chemotherapy, radiotherapy, and surgery. Survival rates depend on histological subtype and stage of disease, with cure rates ranging from 85% among patients with stage I to 10% to 20% for patients with stage IV disease after a combination of local and systemic treatment [3]. Treatment of sarcomas in adolescents and young adults is complex due to age-specific issues that always require to be managed through a multidisciplinary approach to improve global outcomes [4].

Frequently in high-risk cases, the mainstay of systemic treatment consists of intensive alkylating agent-based multidrug chemotherapy [4], which is known to be extremely gonadotoxic. Many female patients treated with chemotherapy and pelvic radiotherapy for a diagnosis of sarcoma experience premature ovarian insufficiency (POI) and impaired fertility later in life as a consequence of treatment [5]. It is estimated that, as a result of gonadotoxic treatments, at least one-third of the female cancer population undergoes POI [6]. As acknowledged by clinicians, most cancer survivors express reproductive concerns and many women who are cured of cancer will develop a desire to have children in the future [7,8]. The most recent guidelines recommend that oncologists address the issue of fertility preservation as soon as possible with their young patients, providing adequate counselling about their risk of subsequent infertility and, if considered safe from an oncological perspective, referring them to a fertility clinic for access to fertility preservation strategies [9]. The most established fertility preservation options in oncology currently include controlled ovarian stimulation (COS) with subsequent embryo or oocyte cryopreservation, and ovarian tissue cryopreservation before the beginning of cancer treatment [9].

Embryo cryopreservation is an option that can be rarely applicable in the setting of fertility preservation in oncological patients, due to ethical constraints in case the partner is no longer available or the patient dies. Thus, the gold-standard approach in this setting is cryopreservation of oocytes. In oncological patients needing to start cancer treatment, oocyte cryopreservation can be achieved in approximately 2 weeks. Due to potential genetic damage to oocytes, it can only be offered before the beginning of chemotherapy [10]. In patients with cancer, to avoid excessive delays in cancer treatment initiation, a random-start protocol, which is independent of the menstrual cycle phase, can be adopted without compromising the efficacy of stimulation [11]. After COS, the final maturation of oocytes is induced and oocytes are retrieved through a transvaginal ultrasound-guided pick-up. In women who need to start treatment urgently, or in pre-pubertal patients who cannot undergo hormonal stimulation, the preferred option is ovarian tissue cryopreservation, which is faster since it does not require prior treatments [12]. This technique was until recently considered experimental by the major societies of reproductive medicine; however, it is now considered an acceptable procedure for fertility preservation by ESHRE guidelines [13], with reported live birth rates around 30–40% in the largest case series [14].

While the impact of chemotherapy and radiotherapy on fertility is an established piece of evidence, with many underlying mechanisms being hypothesized including damage to cycling follicles within the ovary, follicular burn-out, stromal damage, and demise of follicular support cells [6], many authors have also observed that a cancer diagnosis in and of itself might in fact be detrimental on fertility in both sexes [15,16]. Abnormal semen parameters have been found in male patients before the beginning of cytotoxic treatments [15]. A reduced number of retrieved oocytes after COS was found in women with cancer even before receiving any chemotherapy, with respect to healthy patients of the same age [17].

Underlying mechanisms could include the switch to a catabolic state induced by increased stress hormone levels and a hypothalamic dysfunction [18]. It has in fact been hypothesized that, in women, psychological stress can lead to reproductive failure through immune-endocrine disruption [16]. However, this is likely a partial explanation of a more complex phenomenon, since reduced anti-Mullerian hormone (AMH) concentrations and follicle density have also been observed in certain types of cancer before the beginning of cytotoxic treatments, suggesting an impairment of ovarian reserve itself [19]. This impairment of ovarian follicular density could be explained by an increased expression of metalloproteinases or growth factors such as tumor growth factor-beta (TGF-beta) by some cancers [20,21].

Several studies have shown worse ovarian response in cancer patients undergoing hormonal stimulation for oocyte cryopreservation, thus compromising the efficacy of fertility preservation procedures for oncological reasons [17,22]. However, there is still an ongoing debate on this issue, as other studies did not confirm these results [16,23]. The controversial data in the literature could be due to the low number of patients included in published case series and the lack of disease-specific analyses. Evidence appears to be more solid in studies focusing on patients diagnosed with hematological malignancies, where both basal fertility and response to stimulation are reduced with respect to healthy controls [24]. Some data can also be found about a lower response to stimulation in patients with hormone-dependent malignancies such as breast cancer [17]. Instead, data on the performance of patients diagnosed with sarcoma are scant, since currently no published studies are focusing on this population.

The objective of this analysis was to compare the ovarian reserve and outcomes of COS of a population of young women with a diagnosis of sarcoma with those of a control population of women without cancer. This analysis aims to assess whether an impaired response to stimulation exists among patients with a diagnosis of sarcoma, potentially identifying a need for alternative or additional fertility preservation strategies in this population.

## 2. Materials and Methods

This is a monocentric case–control study comparing women undergoing COS for a diagnosis of sarcoma with a control population, receiving COS for isolated severe male factor infertility. After analyzing the distribution of semen values in our assisted reproduction center, we specifically looked at the lowest quartile of values for couples with male factor infertility to determine the threshold for severe male factor infertility. As a result, we set the threshold at less than 0.15 million motile sperm per milliliter of semen. We adopted this approach due to the lack of a universally agreed definition of severe male factor infertility. Women with endometriosis, idiopathic infertility, tubal disease or prior chemotherapy were excluded.

Protocols for gonadotropin stimulation and transvaginal oocyte retrieval were conducted according to standard practice [25]. Ovulation induction was obtained either with 10,000 UI recombinant hCG or 0.2 mg GnRH-agonist when at least three follicles reached a mean diameter of 18 mm, as described previously [26]. Patients with sarcoma underwent ovarian stimulation with a random-start protocol [27] and their oocytes were vitrified after collection for future use [28].

Primary study outcomes included pre-treatment antral follicular count (AFC) and number of retrieved mature oocytes (MII—metaphase II) after stimulation. Secondary study outcomes included the total dose of gonadotropin administered, days of stimulation, and follicle-stimulating hormone (FSH) dose/oocyte ratio.

Sarcoma cases were matched with controls of comparable age (range ± 1.5 years) and year of stimulation (range ± 1.5 years). To facilitate the matching, a standard age of 29 was used for all women under 29 years of age, due to the limited number of women below the age of 29 years in the control group. The database of cases and controls was created using R version 4.1.2, performing a match by group without replacement [29]. Subsequently, univariate comparisons were performed using Mann–Whitney test, and multivariate comparisons using linear regression in SPSS v.27 for Mac (SPSS, Chicago, IL, USA). All calculated *p*-values were two-sided and *p*-values <0.05 were considered significant.

The study was approved by the Institutional Review Board of San Raffaele Hospital (protocol Oncofertility v2.0 n.284/11). Patients signed a written informed consent for the use of personal data.

## 3. Results

Between 2013 and 2021, a total of 84 women with a diagnosis of sarcoma received counselling on fertility preservation at San Raffaele Hospital Oncofertility Unit. Among these, 43 patients underwent COS and oocyte cryopreservation. Table 1 reports reasons for not undergoing oocyte cryopreservation in the cohort of patients with a diagnosis of sarcoma. The most frequent reason for not undergoing fertility preservation procedures was the presence of a clinical contraindication. In those patients where treatment was to be initiated urgently and in pre-pubertal patients where hormonal stimulation was not an option, the cryopreservation of ovarian tissue was preferred. Five patients refused any procedure for personal reasons, and one patient had a failed stimulation that was interrupted due to monofollicular growth.

In patients undergoing COS, histological diagnoses were osteosarcoma (12 patients, 18%), Ewing sarcoma (11 patients, 17%), liposarcoma (8 patients, 13%), synovial sarcoma (5 patients, 8%), rhabdomyosarcoma (4 patients, 6%), chondrosarcoma (3 patients, 5%), and other histologies (21 patients, 33%).

The control cohort included 355 patients undergoing COS for isolated severe male factor infertility.

In the final analysis, a total of 37 patients with sarcoma were matched in a 1:3 ratio with 109 healthy controls due to the lack of adequately matched controls for 6 of the sarcoma patients. In the sarcoma patients’ population, 15 patients out of 37 (40%) were in the luteal phase of menstrual cycle at the beginning of hormonal stimulation.

Table 2 reports the results of univariate comparisons between sarcoma cases and controls, respectively, in the whole population, in the population of women younger than 32 years and in the population of women older than 32 years.

Considering the whole population, patients diagnosed with sarcoma were significantly younger than controls. Body mass index (BMI) was similar between the two cohorts.

In the univariate analysis, the average AFC did not significantly differ between patients with sarcoma and their age-matched controls (13.2 ± 6.9 vs. 12.7 ± 6.1, *p*-value = ns). Significant differences could be observed between the stimulation protocols of sarcoma patients and controls: in particular, lower FSH doses were used in patients cryopreserving oocytes for a sarcoma, compared with patients undergoing COS for assisted reproductive techniques (ART) (1643.7 ± 1168.0 vs. 2001.1 ± 932.0, *p* = 0.007). Different hormone levels were reported on trigger day, with significantly higher levels of estradiol in the control group.

In general, patients with sarcoma did not perform worse than controls during stimulation, with an average metaphase II (MII) retrieval of 10.6 oocytes vs. 8.1 in the control population. Interestingly, they also showed a more favorable FSH dose/oocyte ratio (198.4 ± 200.7 vs. 326.8 ± 389.4, *p* = 0.009).

In the subgroup analysis by age, women below age 32 had a less precise matching due to the higher average age in the control group. This partially explains the higher number of retrieved oocytes in the sarcoma group, composed of younger patients, with respect to controls. Over age 32, instead, where the matching was more accurate, no differences in oocyte retrieval outcomes were observed between sarcoma patients and controls. In this subgroup, the difference in stimulation protocols was more evident, with higher FSH doses and higher levels of estradiol in the control cohort.

In the multivariate analysis, a linear regression was conducted to estimate the difference in the number of retrieved MII oocytes, adjusted for confounding factors. Factors included in the regression were sarcoma diagnosis, AFC and age. Table 3 reports the results in the whole population and in the two subgroups of women under age 32 and over age 32.

The results of linear regression on the number of retrieved mature oocytes showed that patients with sarcoma performed comparably to healthy controls during COS.

## 4. Discussion

Thanks to advances in cancer treatment, survival rates are increasing. The downside of this remarkable achievement is that more women in the future are expected to face the negative consequences of oncological therapies received when they were younger. One of the most important side effects of treatment in the population of young women surviving cancer is infertility, being associated with severe psychological distress and relational issues [8]. Addressing this impacting consequence before the beginning of oncological treatment is a necessary step in global patient care, which entails the identification of the best fertility preservation strategy in each clinical situation. Management of reproductive-aged women with a malignancy requires a multidisciplinary team trained to evaluate the risk of infertility and possible risks and benefits of available fertility preservation techniques. Individual risk of infertility and POI is not easily determined upfront, being the result of a combination of patient-related and treatment-related factors [5].

Although embryo cryopreservation should be considered the most reliable fertility preservation technique according to live birth rates, in cancer patients, due to ethical barriers, oocyte cryopreservation is, to date, considered the gold standard for fertility preservation. The reported live birth rates for oocyte cryopreservation are approximately 35% per stimulation cycle in women aged 30–35 [12]. However, stimulation responses could differ significantly depending on the specific diagnosis. According to some authors, cancer can be detrimental to gonadal function independently from gonadotoxic treatments. Quintero et al., (2010) showed that stimulation in oncological patients requires higher gonadotropin doses [22]. Friedler et al., in their meta-analysis, reported a lower number of oocytes retrieved after COS for fertility preservation in patients with different types of cancer, compared with age-matched healthy controls [17]. Several factors could contribute to the impairment of reproductive function, including malnutrition, an increase in stress hormones such as prolactin, or the secretion of cytokines by cancer tissue [18]. Whichever the mechanism behind this phenomenon, it is reasonable to hypothesize that all of these factors could impact in a significantly different way according to cancer histotype.

Evidence of a reduced basal fertility in patients with a diagnosis of lymphoma can be found in the literature [24,30]. Men diagnosed with lymphomas have shown to have decreased sperm quality [15] and female patients undergoing COS after a diagnosis of lymphoma appear to have a similar gonadal dysfunction. In particular, in the study by Lekovich and colleagues [24] women with lymphomas had lower baseline AMH levels and AFC when compared to healthy controls and women with a different type of malignancy; moreover, their oocyte retrieval was reduced with respect to controls after adjusting the results by age. These findings are similar to those reported by Lawrenz et al. in another case series of female lymphoma patients [30].

While the performance of patients with hematological malignancies has been investigated in dedicated case series, currently there are very few data on the performance of pre-treatment COS in young patients being diagnosed with a sarcoma.

In the study by von Wolff et al. (2018), COS outcomes of patients suffering from different types of cancer were analyzed and compared to a control population of breast cancer patients: a total of 37 patients with a sarcoma were included, with an average oocyte yield of 13 [23]. Another case series presented stimulation data of a population of 11 patients with soft tissue sarcoma, where a mean of 18 aspirated oocytes was reported [16]. Both case series conclude for no detrimental effect on fertility for all types of cancer considered.

In our analysis, we sought to exclusively evaluate the performance of sarcoma patients on COS by comparing their recovery outcomes with those of a presumptive healthy control population. The results showed that patients with a sarcoma have performances comparable to those of patients who refer to a fertility center exclusively due to the severe male factor of the partner. Notably, sarcoma patients obtained an average of 1.21 more MII oocytes than age-matched controls. Based on the confidence intervals calculated in the logistic regression, we can estimate that in the worst-case scenario, the sarcoma patients achieve, at most, a reduction of 0.82 MII oocytes. This represents a 9.6% maximum estimable reduction compared to the average number of MII oocytes retrieved from controls. Even limiting the analysis to patients over age 32, where the matching is more accurate, the result remains the same. In fact, in this subgroup, the patients with sarcoma recovered an average of 0.37 MII oocytes more than the controls. Therefore, the estimates obtained from our analysis indicate the absence of clinically relevant differences. A fertility specialist should then expect an ovarian response after COS similar to that of patients of similar age and ovarian reserve, with no particular diagnosis of female infertility. Our results are, therefore, reassuring on the outcomes of COS in sarcoma patients, who are generally young and expected to receive intermediate-to-highly gonadotoxic chemotherapy schedules [31].

Regarding ovarian stimulation protocols, both sarcoma patients and controls were subjected to standard protocols. The standard protocol for fertility preservation in patients with cancer includes stimulation with a gonadotropin releasing hormone (GnRH) antagonist protocol, and final maturation induction with a GnRH agonist, which is known to reduce the risk of ovarian hyperstimulation syndrome (OHSS) compared to a human chorionic gonadotropin (hCG) trigger [32]. It should be considered that patients with sarcomas underwent ovarian stimulation with a random-start protocol, which has proven to be advantageous in terms of time required for stimulation in emergent situations, without a significant impact on oocyte yield and maturity [27]. Patients in the control group were, instead, stimulated with a conventional start protocol, beginning in the early follicular phase, and received either GnRH agonist or hCG as a trigger. Almost half of the sarcoma patients began stimulation in the luteal phase: this could justify the average lower estrogen levels reported on the trigger day. Additionally, it is worth mentioning that patients with sarcoma have been stimulated with particular attention to reducing the risk of complications due to hyperstimulation, in order to avoid possible delays in cancer treatment initiation. This could explain why the ovarian stimulation data showed lower total FSH dose values for cancer patients compared with controls. In this respect, patients with a sarcoma showed a better performance in terms of FSH dose/oocyte ratio, which was found to be significantly lower than controls. However, the difference in the FSH/oocyte ratio was not significant in the over age 32 subgroup, suggesting that it may be partly due to the age discrepancy observed in matching younger patients.

The strengths of our analysis include the fact that both cases and controls were treated at the same center, and the strict selection of controls and their matching by age and year of stimulation, which provides reliable comparisons between the groups. Conversely, study limitations include the less precise matching of patients aged less than 32, due to a generally older control population. However, the latter should not impact the general results of the analysis, as in terms of response to COS, age differences are less pronounced in women aged under 30 when the ovarian reserve is bigger.

Finally, another possible limitation of the study is the selection of the healthy control group in the setting of ART. Given the lack of a clinical diagnosis of infertility in the female partners and the severity of the male factor, the population undergoing COS for severe male factor infertility was presumed to be free from other known fertility-reducing conditions. However, despite the careful selection of these controls and the matching by age, we could not be able to rule out an intrinsic bias, since these patients may not be fully representative of a fertile control population.

## 5. Conclusions

In summary, we can conclude from our data that patients with sarcoma can expect good egg retrieval outcomes after COS, even with a random-start protocol and the use of sub-maximal doses of gonadotropins. This should further encourage oncologists to address young patients with sarcoma to a fertility preservation facility whenever possible and safe from an oncological perspective, to overcome the burdensome issue of treatment-related infertility. Fertility preservation should be integrated into cancer care to improve the outcomes and future quality of life of survivors.

## Figures and Tables

**Table 1:** Reasons for not undergoing oocyte cryopreservation in patients with a diagnosis of sarcoma.

Reason for Missed Oocyte Cryopreservation	Number of Patients
Clinical contraindication	7
Already started chemotherapy	2
Patient choice	5
Failed stimulation due to monofollicular growth	1
Ovarian tissue cryopreservation preferred	24
Unknown reason/patient lost at follow up	2

**Table 2:** Characteristics of patients with a sarcoma and healthy controls (whole population, women below 32 years of age and women over 32 years of age). All the results are expressed as mean +/− standard deviation. BMI: body mass index; AFC: antral follicular count; FSH: follicle-stimulating hormone; IU: international units; MII: metaphase II.

	Controls (N = 109)	Sarcoma (N = 37)	*p*-Value	Sarcoma ≤ 32 Years (N = 18)	*p*-Value	Sarcoma > 32 Years (N = 19)	*p*-Value
Age (years)	32.8± 3.5	29.4 ± 5.9	0.001	24.2 ± 2.9	0.0001	34.3 ± 3.1	0.600
BMI (kg/m^2^)	21.4 ± 3.2	21.4 ± 3.1	0.975	21.2 ± 2.9	0.294	21.7 ± 3.4	0.355
AFC (n)	12.7 ± 6.1	13.2 ± 6.9	0.670	14.7 ± 7.9	0.819	11.7 ± 5.7	0.660
Days of stimulation (n)	9.7 ± 1.9	10.3 ± 2.0	0.155	9.6 ± 2.0	0.594	10.8 ± 1.9	0.022
FSH total dose (IU)	2001.1 ± 932.0	1643.7 ± 1168.0	0.007	1510.7 ± 1359.0	0.120	1769.7 ± 974.6	0.017
Estradiol on trigger day (pg/mL)	2078.1 ± 1147.5	1331.9 ± 835.9	0.0001	1525.4 ± 1035.3	0.054	1138.4 ± 543.6	0.001
Progesterone on trigger day (ng/mL)	0.7 ± 1.2	0.8 ± 1.0	0.534	0.8 ± 1.2	0.329	0.7 ± 0.9	0.975
Retrieved oocytes (n)	10.3 ± 5.8	13.0 ± 8.3	0.147	16.3 ± 8.9	0.011	9.8 ± 6.6	0.691
MII oocytes (n)	8.1 ± 5.2	10.6 ± 7.1	0.064	13.0 ± 7.6	0.009	8.3 ± 5.9	0.909
FSH dose/oocyte ratio	326.8 ± 389.4	198.4 ± 200.7	0.009	126.4 ± 157.7	0.005	262.8 ± 216.7	0.210

**Table 3:** Results of linear regression analysis of the number of MII oocytes in patients with sarcoma and controls corrected by age and AFC in the whole population and in the 2 age subgroups (below and over 32 years). MII: metaphase II. CI: confidence interval.

	Coefficient (Estimate of MII Difference in the Group with a Sarcoma) *	CI 95%	*p*-Value
Whole population	1.206	−0.824–3.235	0.242
Age ≤ 32 years	2.720	−2.122–7.562	0.266
Age > 32 years	0.371	−1.994–2.736	0.755

* Corrected by age and antral follicular count.

## Data Availability

The data presented in this study are available on request from the corresponding author. The data are not publicly available due to privacy restrictions.
